# A randomized double blinded placebo controlled study to evaluate motor unit abnormalities after experimentally induced sensitization using capsaicin

**DOI:** 10.1038/s41598-021-93188-7

**Published:** 2021-07-02

**Authors:** Valerie Evans, Ryan G. L. Koh, Felipe C. K. Duarte, Lukas Linde, Mohammadreza Amiri, Dinesh Kumbhare

**Affiliations:** 1 Toronto Rehabilitation Institute, University Health Network, Toronto, ON Canada; 2 Department of Medicine, Institute of Biomaterials and Biomedical Engineering (IBBME), University of Toronto, 550 University Ave, Suite 7-131, Toronto, ON M5G 2A2 Canada; 3 Division of Research and Innovation, Canadian Memorial of Chiropractic College, Toronto, ON Canada; 4 International Collaboration on Repair Discoveries, University of British Columbia, Vancouver, BC Canada; 5 School of Kinesiology, University of British Columbia, Vancouver, BC Canada; 6 Division of Physical Medicine and Rehabilitation, Department of Medicine, University of Toronto, Toronto, ON Canada

**Keywords:** Physiology, Medical research, Engineering

## Abstract

Central sensitization is a condition that represents a cascade of neurological adaptations, resulting in an amplification of nociceptive responses from noxious and non-noxious stimuli. However, whether this abnormality translates into motor output and more specifically, ventral horn abnormalities, needs to be further explored. Twenty healthy participants aged 20–70 were randomly allocated to topical capsaicin or a placebo topical cream which was applied onto their left upper back to induce a transient state of sensitization. Visual analogue scale (VAS) ratings of pain intensity and brush allodynia score (BAS) were used to determine the presence of pain and secondary allodynia. Surface electromyography (sEMG) and intramuscular electromyography (iEMG) were used to record motor unit activity from the upper trapezius and infraspinatus muscles before and twenty minutes after application of capsaicin/placebo. Motor unit recruitment and variability were analyzed in the sEMG and iEMG, respectively. An independent t-test and Kruskal–Wallis H test were performed on the data. The sEMG results demonstrated a shift in the motor unit recruitment pattern in the upper trapezius muscle, while the iEMG showed a change in motor unit variability after application of capsaicin. These results suggest that capsaicin-induced central sensitization may cause changes in ventral horn excitability outside of the targeted spinal cord segment, affecting efferent pathway outputs. This preclinical evidence may provide some explanation for the influence of central sensitization on changes in movement patterns that occur in patients who have pain encouraging of further clinical investigation.

Clinical Trials registration number: NCT04361149; date of registration: 24-Apr-2020.

## Introduction

Chronic pain is characterized by pain lasting longer than three months^1–3^. It is a common and disabling condition affecting 1 in 5 people^4^ and is rising due to the aging population and increased prevalence of comorbid conditions such as diabetes^5,6^ and obesity^7^. Chronic pain produces a significant socioeconomical burden with the annual estimated cost in Canada exceeding 6 billion dollars^4,8–11^. As such, the assessment and diagnostic efficacy of chronic pain is of vital importance to improving its management throughout society.

Central sensitization describes a state of neuronal hyper-excitability in the central nervous system that may occur due to malfunction of spinal and supraspinal pain facilitatory and inhibitory circuits resulting in amplification of somatosensorial responses^12^. Beyond somatosensorial changes, alteration in motor function can also be present with pain and may be an impairment of neuromuscular function^13,14^. A normal afferent input and normal central processing circuitry is essential to deliver normal efferent output. However, the influence of the changes that occur within the dorsal horn on the ventral horn remain largely ill-defined^13,15^.

Motor unit assessment is crucial in evaluating diseases and abnormalities within the ventral horn^16^. Activity of the ventral horn, where anterior horn cells reside, is very important for motor unit activation^17^. Surface EMG (sEMG)^18^ and intramuscular EMG (iEMG)^19^ can be used to assess the neural drive to muscles, by recording motor units to understand the effects of central sensitization on motor control and the ventral horn. Based on Henneman’s size principle, motor units should be recruited in the same order, with smaller units being recruited first^17^. Evaluation of this principle presents an opportunity to investigate if central sensitization creates abnormalities at the motor unit level.

Previously, central sensitization has been induced in healthy subjects to examine its neurophysiological effects via capsaicin^20,21^. Capsaicin, a chili pepper extract, has become the accepted method to effectively induce experimental transient states of central sensitization^20–24^. The presence of expanded sensorial responses and the involvement of the spinal nociceptive system post capsaicin have been largely tested by means of quantitative sensory testing methods and electromyography (EMG)^20,21,23,24^. Despite the usefulness of experimental capsaicin to better understand the sensorial abnormalities, its impact on motor function and motor unit recruitment are lesser studied or understood. Evidence suggests that nociceptive input by peripheral capsaicin exerts a centrally-mediated inhibitory effect on motor function^25^. A decrease in root mean squared (RMS) amplitude during exercise at the time of peak sensitization was measured by needle EMG^25^. However, the effect that capsaicin-induced sensitization has on individual motor units or on their recruitment patterns has not been previously examined.

The purpose of this study was to determine whether topical capsaicin-induced sensitization has any influence on anterior horn cell activity. We hypothesize that capsaicin induces a change in individual motor unit activity, as well as the recruitment pattern of many motor units, and may affect motor unit activity at different segmental levels from the level of capsaicin application. We would expect to observe an effect within the same neuromeric segment however, since anatomically, there are tracts (tracts of Lissauer) that link the affected spinal segment to the segments above and below^26^. As a secondary outcome we wanted to explore whether topical capsaicin application would influence these distal spinal segments.

## Methods

### Design overview and study participants

All participants provided written informed consent before participating in this double-blinded, randomized, parallel group, placebo-controlled study. The protocol approval was obtained from the Ethics Board of the Toronto Rehabilitation Institute, University of Toronto prior to commencing the study (REB#: 18-6163). This study was conducted in accordance to the World Medical Association Declaration of statement of ethical principles for medical research involving human subjects^27^. This study recruited men and women aged ranging between 20 and 70 years old from the general population using online postings and word-of-mouth. Additional information can be found on ClinicalTrials.org, using the identification number NCT04361149. This trial was registered on April 24, 2020.

#### Inclusion and exclusion criteria

For inclusion, participants had to be asymptomatic with symptom-free of pain and musculoskeletal disorder in the last three months or presenting low pain severity below 30 mm in the pain visual analogue scale (VAS)^28^. Since prevalence of neck pain in the general population is high, mild pain or aches are not necessarily related to an abnormality of the underlying muscle^3^. Also, included subjects had to have a self-reported normal body mass index (18.5–24.9)^29^ and had to be able to communicate in English.

**Figure 1:**
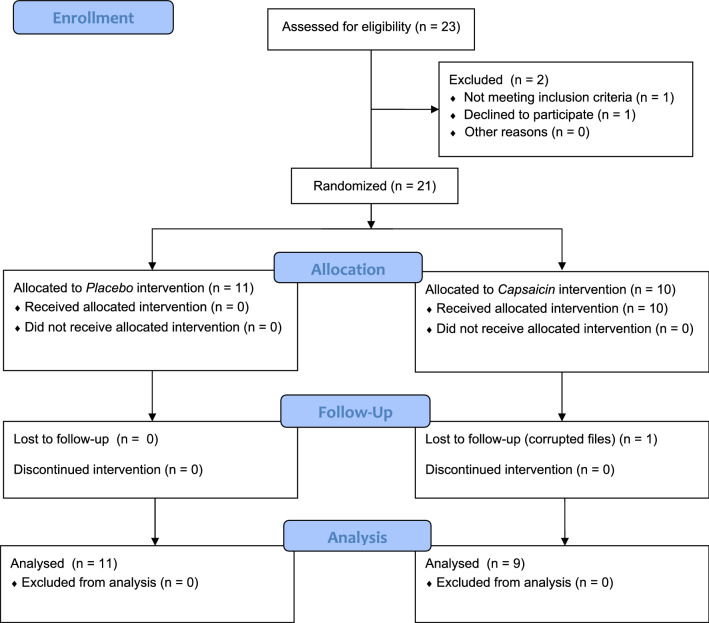
PRISMA flow chart.

Participants were excluded whether presenting history of direct trauma to cervicothoracic region, medical history of inflammatory disorders as rheumatoid arthritis, neurodegenerative disorders such as Parkinson's disease and motor neurone diseases as amyotrophic lateral sclerosis, or any other neuromuscular disorder. Participants were excluded if they had persistent pain for more than 3 months. This was determined by the physician on our research team (DK). See Table 1 and Fig. 1 for participant information and the CONSORT flowchart respectively.

**Table 1:** Subject and brush allodynia characteristics.

Participant (N = 23)	Gender	Treatment (placebo or capsaicin)	Age	Visual analogue score (VAS) 0–100 mm		Difference of areas of mechanical allodynia between pre and post treatment (cm^2^)	Issues
				Pre	Post 20 min		
1	M	Placebo	61	0	0	0	–
2	F	Placebo	57	0	20	0	–
3	F	Placebo	61	0	0	1.0	–
4	M	Capsaicin	29	0	37	195	–
5	M	Capsaicin	66	0	45	348.75	–
6	F	Placebo	27	–	–	–	Withdrew
7	M	Capsaicin	20	0	60	0	–
8	F	Capsaicin	21	–	–	–	Corrupted files
9	F	Placebo	27	0	0	0	–
10	F	Capsaicin	25	0	70	256	–
11	F	Capsaicin	24	0	20	222	–
12	F	Placebo	27	0	0	0	–
13	M	Capsaicin	35	0	70	58.5	–
14	M	Placebo	27	0	20	0	–
15	F	Capsaicin	23	0	80	76	–
16	F	Placebo	22	30	30	0	–
17	F	Capsaicin	22	0	60	12	–
18	M	Placebo	34	0	0	0	–
19	M	Capsaicin	61	–	–	–	Did not meet inclusion criteria
20	M	Capsaicin	27	0	50	104	–
21	F	Placebo	24	10	10	0	–
22	M	Placebo	30	0	0	0	–
23	F	Placebo	30	0	0	0	–

#### Outcomes

The primary outcomes were the motor unit activity such as motor unit recruitment and motor unit variability measured by surface electromyography (sEMG) and intramuscular electromyography (iEMG), respectively from the upper trapezius and infraspinatus muscles.

#### Sample size calculation

The minimum sample size required per group to detect whether a significant difference exists between two means for one dependent variable was calculated with a confidence level of 95%, an 80% statistical power level and 5% error. A mean detectable (and clinically relevant) difference of 5 for the dependent variable, mean motor unit recruitment change, and an estimated standard deviation of 3 was used. The estimated sample size was 7 people per group; however, considering a 20% drop out rate, the suggested sample size was 9 people per group. This analysis was completed using PASS Software, version 15.

#### Randomization and group allocation

For randomization and group allocation, recruited participants were randomized in one of the two groups: Capsaicin group and skin lotion (placebo). Randomization and group allocation was given by using a random number generator (using a computer) before testing that it was performed by an investigator not involved in the data collection (MA). Opaque envelopes containing the treatment (capsaicin x placebo) and study subject numbers were prepared in advance and handed to one of the study investigators (FD) who was responsible for capsaicin or placebo cream application during the participant testing session day. All participants were not told their group allocation. None had received capsaicin cream previously, so they did not have any preconceived bias. Despite this we acknowledge that capsaicin cream gives visual clue as redness and burning pain sensation which may allow the participants to recognize the group they belong. Most significantly, the participant would not know what EMG and ultrasound (US) data was being collected and how the capsaicin would influence them. Also, investigators involved in the data collection (VE, DK) were not informed of the treatment group to which the participants belonged.

### Pain assessment, sensory testing and dataset collection

To determine eligibility for the study, an initial screening of each potential study participant was performed by applying the inclusion and exclusion criteria of the study. Each participant was seated upright with their hands comfortably on their lap and asked to relax their neck and shoulder muscles. The physician member of the research team then assessed the participants’ pain intensity by visual analogue scale (VAS). VAS ranges from 0 to 100 mm which 0 mm reflecting no pain at all and 100 mm representing the worst imaginable pain^28^. Following this, brush allodynia, a clinical technique used to identify pain due to a stimulus that does not normally provoke pain, was performed to confirm presence of central sensitization^12^. To map out borders of secondary allodynia, subjects were instructed to recognize a distinct alteration in the sensation perception such as increased burning, intense pricking, or an unpleasant sensation, and that location was marked^30^. Brush allodynia score (BAS) was calculated as the distance between the farthest points marked on the superior and inferior axis multiplied by the distance between the farthest points marked on the medial and lateral axis as previously described by Cavallone et al.^30^. BAS is typically the area of increased mechanical allodynia including the area that the capsaicin was applied. The presence of pain central sensitization by means of VAS and BAS were assessed at baseline (pre) before the induction of sensitization and twenty minutes after (post). The criteria for determining whether central sensitization has been achieved included one of an increase in the area of mechanical allodynia and a significant increase in the VAS. Please see “Application of intervention” section below for more detailed information of intervention.

**Figure 2:**
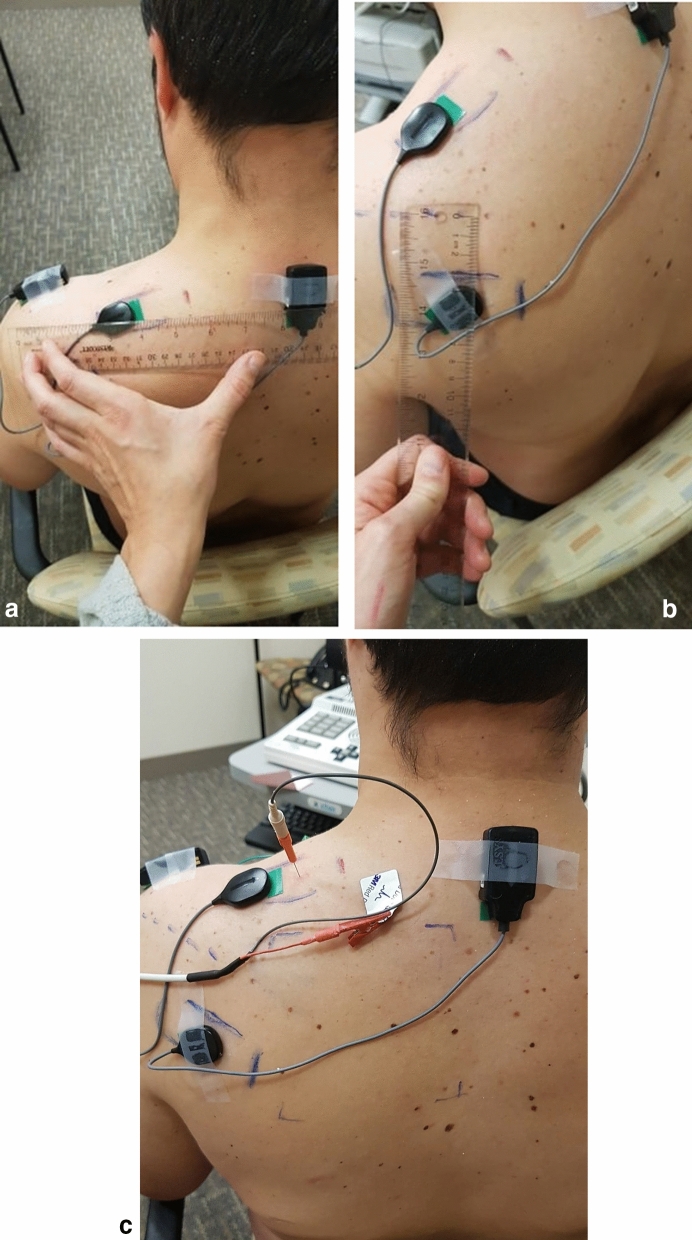
(**a**, **b**) Surface electromyography electrode placement on upper trapezius muscle and infraspinatus. (**c**) Intramuscular needle electrode placement on trapezius muscle.

Upon successful screening of inclusion and exclusion criteria, participants had their left side area of skin (overlying the upper trapezius and infraspinatus muscles) cleansed with alcohol preparation pads and water. The skin was abraded with ‘3 M Red Dot’ abrasive strips before application of the surface electromyogram (Trigno Galileo sensors, *Delsys Inc*.^31^). Electrodes were placed in 4 areas: the muscle belly of the upper trapezius, and the infraspinatus muscles, as well as reference electrodes on C7 and the acromion (Fig. 2a,b). These electrodes were 4 channel EMG sensors and had their signals filtered from 20 to 450 Hz. The sEMG recordings were wirelessly transmitted to the Trigno base station, which relays and compiles the data to Neuromap (*Delsys Inc.*) for signal analysis^31^. A monopolar needle electrode was inserted into the upper fibers of trapezius muscle and its reference was placed at the mid-clavicle point. Using this setup intramuscular recordings of single motor units were performed using an XCalibur, *Natus Medical* clinical electrodiagnostic machine^32^*.*

#### Electromyograms recordings before application of intervention

Participants were instructed to perform horizontal shoulder abduction from 0° to 90° and then adduction from 90° to 0° repeatedly for 1 min. The study subject was verbally cued to move their arm every 2 s. sEMGs were recorded during this entire time. Upon completion of this task, a monopolar intramuscular needle electrode was placed directly into the upper trapezius muscle (Fig. 2c). Participants were then instructed to gently contract their trapezius muscle by shrugging their shoulder, enough to recruit only the first motor unit recorded from the region of the intramuscular needle tip. Visual feedback of the signal was given to the participant to ensure that only the first motor unit was activated for the movement. The signal from this motor unit was then optimized by needle movement (very minimal) to hone initial deflection from baseline and amplitude characteristics before recordings were made. iEMG recordings were recorded for 30 s at a sampling frequency of 6 kHz.

#### Application of intervention

Participants received either a dose of 2.5 ml (75 µg/ml) capsaicin cream (0.075% Zostrix, New York, USA) or skin lotion (placebo) which was inert and caused no sensitization effects. Participants were also blinded to the delivered treatment, using concealed containers for the creams. The location of application was a 10 cm by 10 cm square on trapezius muscle which extended from T3 to T8 on the left side that all recordings were conducted.

**Figure 3:**
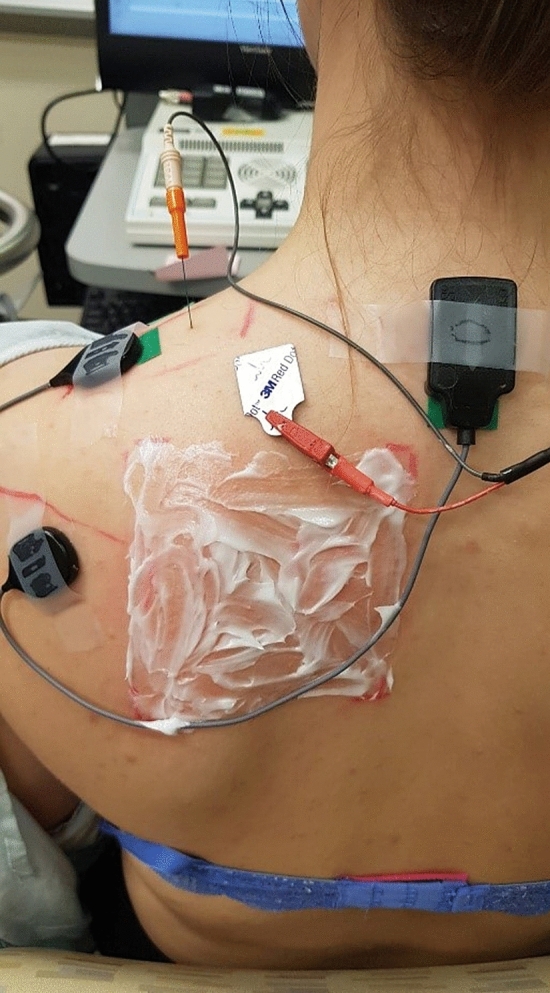
Location of the area of 10 cm by 10 cm application of topical placebo cream or capsaicin onto the skin.

After collection of the baseline VAS, BAS, sEMG and iEMG recordings, a trained medical professional applied the capsaicin/placebo cream directly to the region of skin in a standardized 10 cm × 10 cm square at the spinal levels T3-T8, as seen in Fig. 3, to sensitize the nociceptive afferents within that region. A twenty-minute waiting period was used to enable the sensitizing effects of capsaicin to take effect.

To confirm the presence of central sensitization, VAS and BAS were used to detect mechanical allodynia outside the region of the primary nociception—region of topical placement—which is the region of secondary allodynia^33^. Upon confirmation of central sensitization, in participants with application of topical capsaicin, participants were entered into the experimental arm of the study.

#### Study procedures for EMG analyses

This section outlines the procedures for the EMG analysis. For the sEMG data, the pre- recording motor units were matched with an algorithm implemented in MATLAB (Mathworks, version 2018a), described in “Surface EMG” section and available upon request, in order to determine the aberration of recruitment pattern after treatment.

For the iEMG data, the pre- and post-recording motor units were also matched using an algorithm implemented in MATLAB (Mathworks, version 2018a), described in “Intramuscular EMG analysis using template matching” section. The variability of that motor unit shape was compared before and after capsaicin and between capsaicin and placebo groups.

#### Surface EMG

Recorded sEMGs were analyzed in *Neuromap*^31^, which provides an average template, average amplitude, and order of recruitment of each motor unit detected and isolated from the raw signal using *Delsys*’ motor unit decomposition algorithm^34^. Each motor unit template was then analyzed based on their “overall match” in shape and amplitude, see below.

#### Shape analysis

**Figure 4:**
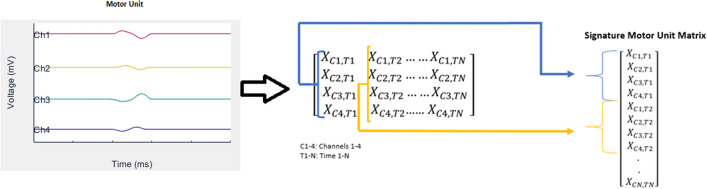
Process of creating the motor unit signature from a set of recordings. Each column of the matrix corresponds to the recordings from all electrode contacts at a single time step up to the time *T*. The columns are then concatenated to create a single vector corresponding to motor unit signature.

The motor unit signal obtained from *M* electrode contacts at *T* consecutive time samples was assembled into an *M x T* matrix. This matrix was then compressed into a single vector to create a motor unit “signature”^35^. This was done by taking the recordings from all electrode contacts at a single time step and concatenating it to the recordings from all contacts at the next time point. This concatenation was repeated to the time *T* to create the signature (Fig. 4). This process was performed for the pre- and post-recording motor units for each participant. In this study, the sEMG motor unit signature was created from 4-electrode contacts × 0.45 ms.

The shape of each detected pre-recording motor unit was compared to the shape of each post-recording motor unit via the cross-correlation function, *xcorr*, in *Matlab* (Mathworks, 2018a)^36^. This function returns a normalized value between 0 and 1 between each pre- and post-recording motor unit. Cross-correlation values between pre- and post-recording motor unit pairs that were below a preset threshold of 0.8^37^ were set to 0 as the pair was unlikely to be from the same motor unit.

#### Amplitude analysis

The motor unit average amplitudes obtained from the *Neuromap* software was used for calculating an amplitude ratio between pre- and post-recording motor units. This amplitude ratio was calculated for each pre/post motor unit pair to determine similarities in the amplitude. Similar to the shape analysis, amplitude ratios that were below a preset threshold of 0.8 or above 1.2 were set to 0. This ratio was normalized to be within a scale from 0 to 1 to allow for comparison between shape and amplitude using the following equation (Eq. 1) when the amplitude ratio was above one.

1 $$Normalized\;Amp.\;Ratio = 1 - Amp.\;Ratio$$

The numbers from the shape analysis and amplitude analysis, which were not set to 0, were averaged together to express an “overall match” between pre- and post- motor units from each participant. As mentioned, based on Henneman’s size principle, motor units should be recruited in the same order with smaller units being recruited first and subsequent motor units are recruited as more force is needed^17^. To observe whether this principle was upheld or violated, the reorganization of the recruitment was identified by how much earlier or later the similar motor units were recruited. In order to quantify the recruitment order the difference in the order of recruitment between the pre and post recording was calculated. Recruitment order of motor units was determined through a custom automated search algorithm (Algorithm 1, Appendix A), which determined the best match between pre- and post-recording motor units.

The average recruitment order difference for each motor unit was obtained for each participant. Following this, for each participant, the recruitment difference for all of their motor units were averaged, to obtain one vector of average recruitment changes per person. This final matrix contained numbers on a continuous scale and was used for statistical testing described in “Surface EMG” section. See Appendix B for a detailed explanation of the matrix set up. For the infraspinatus recordings, in many participants, a low number of motor units were detected. In particular, less than 6 motor units were identified in 7 participants, and no motor units were detected in another 7 participants. As a result of the small sample size, the shape analysis threshold for the infraspinatus muscle was observed at a reduced threshold (0.7) and wider amplitude margin (0.7–1.3) to help observe any underlying trends. This was performed in order to identify more motor units. If this reduced threshold case showed trends closer to those observed in the trapezius case, this can be attributed to a sampling issue.

#### Intramuscular EMG analysis using template matching

Recorded iEMGs were thresholded using the median absolute deviation estimate approach (Eq. 2) to find the peak location of motor unit action potentials (MUAPs)^38^.

2 $$Threshold = ~10 \times \frac{{Median\left( {\left| X \right|} \right)}}{{0.6745}}$$

Each detected peak, along with 12.5 ms before and after was stored and used for analyses. These detected MUAPs were then clustered to determine the number of motor units present in the recording. In particular, the first detected peak and its surrounding environment (i.e. the MUAP) forms the first motor unit template. Subsequent detected MUAPs were compared to the first template using cross correlation. If the cross correlation was more than 0.85, it was determined as the same MUAP and added to that motor unit cluster and the template was updated. However, if the cross-correlation was less than 0.85, it was classified as a different motor unit and a new template cluster was created for comparison.

**Figure 5:**
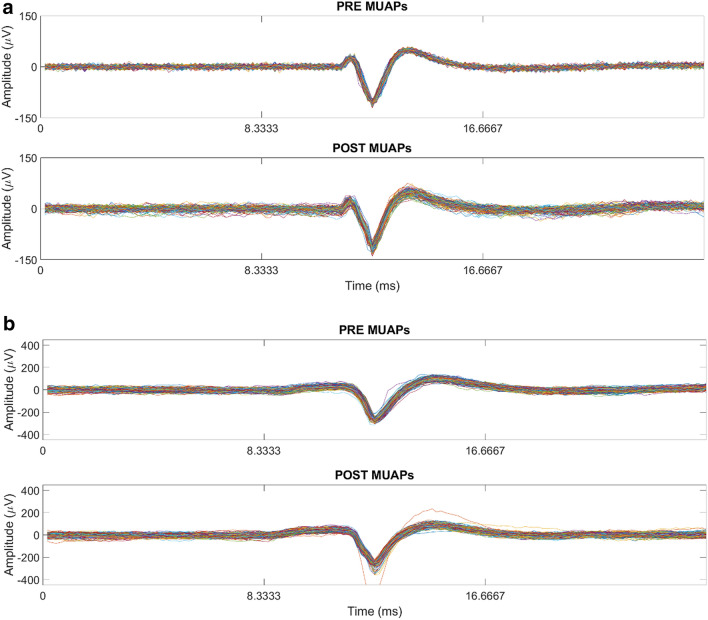
(**a**) Example of motor unit action potentials detected pre and post-recording with the capsaicin treatment. Cross correlation = 0.995. (**b**) Example of motor unit action potentials detected pre and post-recording with the placebo condition. Cross correlation = 0.974.

This process was repeated until all of the detected MUAPs in the pre- and post-recording were classified. The MUAP cluster with the highest number of MUAPs, for the pre-recording, was compared with all MUAP clusters found in the post-recording for each participant. The MUAP cluster in the post that had the highest cross-correlation value was considered “matched.” Examples of matched MUAPs can be seen in Fig. 5. Participants that did not have matched MUAPs with cross-correlation values above or equal to 0.95 were not used for statistical analysis.

### Statistical methods

#### Surface EMG

From the surface EMG algorithm mentioned in “Surface EMG” section, the resultant difference matrix for the trapezius muscle was normally distributed. The differences in recruitment based on size and shape were analysed to determine abnormalities in recruitment. An independent samples t-test was performed on the difference in recruitment position using treatment as the factor for the trapezius muscle. In particular, the statistical test compared post–pre capsaicin recruitment differences against post–pre placebo recruitment differences. The average difference in recruitment order was used to avoid bias of having multiple data points per participant that correspond to different motor units. Assumptions of normality and homogeneity of variances were verified from the Shapiro- Wilk test (*p* = 0.187) and Levene’s Statistic (*p* = 0.081).

For the infraspinatus muscle, the number of samples was much lower, and because of this, the data was not able to achieve normality, and therefore the Kruskal–Wallis H test was used (see Table 2 for group statistics). The statistical test compared the same groups (i.e. post–pre capsaicin recruitment differences against post–pre placebo recruitment differences) mentioned above.

**Table 2:** Significance values of Kruskal–Wallis H test in iEMG.

Condition	Significance
Placebo POST versus Placebo PRE	0.333
Capsaicin POST versus Capsaicin PRE	0.827
Capsaicin POST–PRE versus Placebo POST–PRE	0.043

#### Intramuscular EMG

From the intramuscular EMG algorithm mentioned in “Intramuscular EMG analysis using template matching” section, the resultant matrices were the variances for the pre-recording motor unit trains, and post-recording motor unit trains. The statistical model was designed to assess the difference between the pre- and post- recording motor units for each capsaicin and placebo as well as a between group comparison (Post–Pre capsaicin motor unit variance vs. Post–Pre placebo motor unit variance). These analyses would provide insight into our hypothesis that sensitization induced by capsaicin effects upon the motor unit characteristics.

The variance was calculated from each of the matched MUAP clusters for each participant’s pre- and post-recording at every time point. The variances calculated from all time points (i.e. 150 time samples) were then averaged to get a single average variance value for each participant. A Kruskal–Wallis H test was then performed between the variances found post- vs pre-recording for the two treatments (Capsaicin, Placebo). The Kruskal–Wallis H test was also performed on the difference of the variances found post- vs pre-recording between the capsaicin and placebo treatment. For these statistical tests, four placebo and four capsaicin participants met the criteria of having matched MUAPs with cross-correlation values 0.95 or higher.

## Results

### Confirmation of central sensitization

From the 23 participants recruited, 10 Males and 13 Females, one subject did not meet the inclusion criteria, one subject opted out of the study early, and another subject’s files were not usable due to file corruption (see Fig. 1 for flow diagram for participant enrollment). Table 1 presents the study participant’s characteristics, VAS and area of sensitization outside the area of direct application (BAS). From all nine remaining subjects exposed to capsaicin, only one of the study participants did not achieve changes in BAS after 20 min. However, despite no changes for BAS, this subject presented a meaningful increase for the VAS after application of topical capsaicin. Hence, this subject remained in the data analysis procedures. From Table 1, the average and standard deviation values of VAS for the capsaicin group were pre (0 mm ± 0 mm) and post (54.66 mm ± 18.70 mm) while the values for the placebo group were pre (3.63 mm ± 4.71 mm) and post (7.27 mm ± 5.10 mm). The difference in area of BAS between pre and post for the capsaicin group was 141.36cm^2^ ± 119.78 cm^2^, while the difference in area of BAS for the placebo group was 0.091cm^2^ ± 0.30 cm^2^ (*p* < 0.001, Kruskal–Wallis).

### Surface EMG

**Figure 6:**
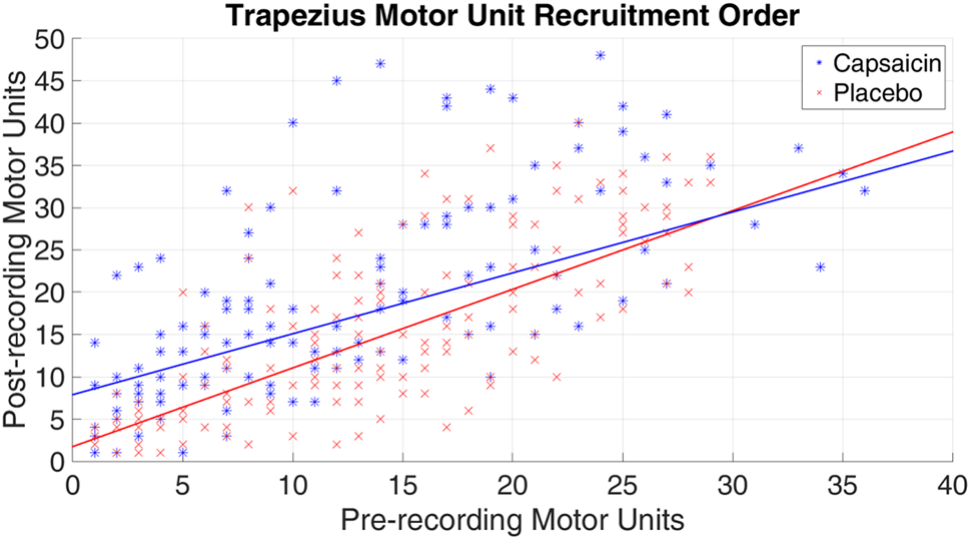
Pre- and post-recording motor units recruitment order in the trapezius muscle. A slope of 1 would correspond to each pre-recording motor unit being recruited in the same order in the post-recording. Shape analysis threshold = 0.8 and amplitude margin = 20%. Capsaicin: R^2^ Linear = 0.464, y = 0.72x + 7.88; Placebo: R^2^ Linear = 0.589, y = 0.93x + 1.74.

**Figure 7:**
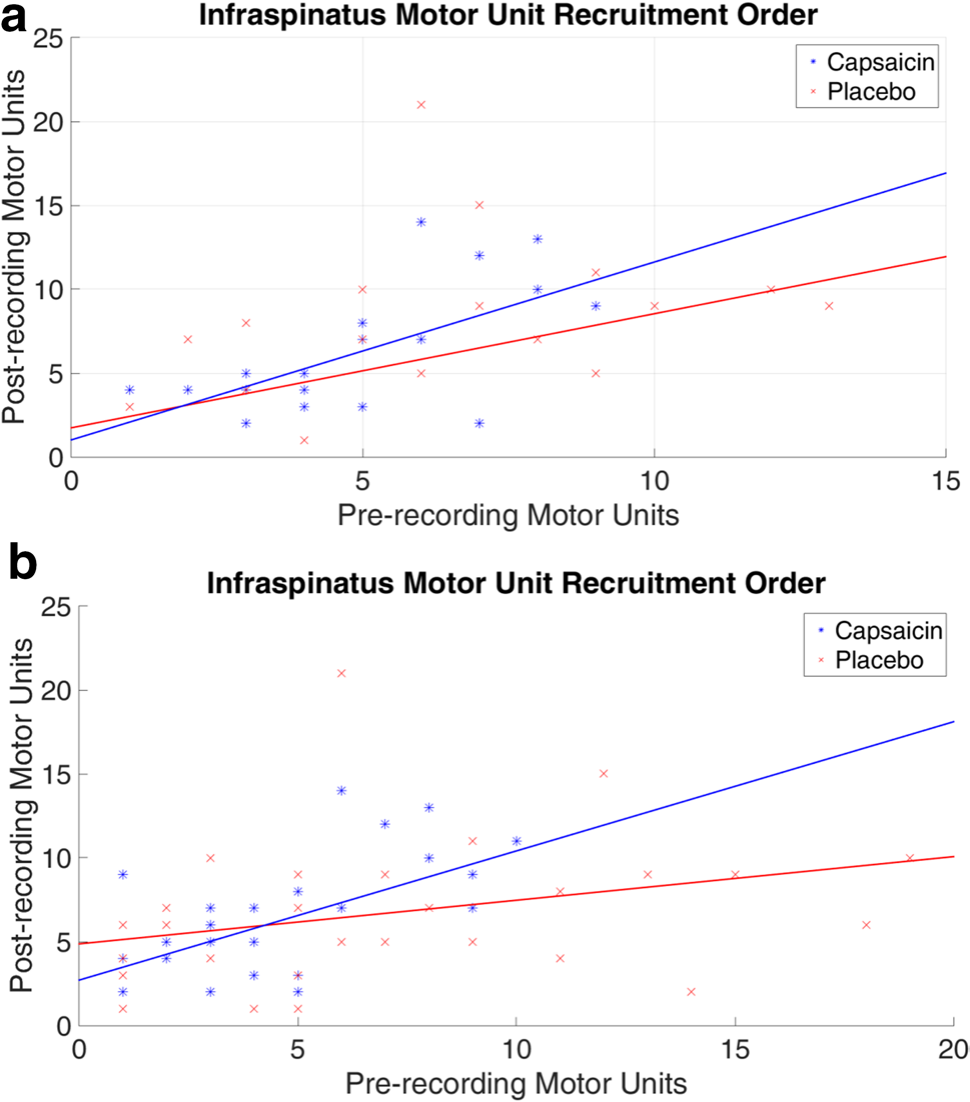
(**a**) Pre- and post-recording motor unit recruitment order in the infraspinatus muscle. Shape analysis threshold = 0.8 and amplitude margin = 20%. Capsaicin: R^2^ Linear = 0.402, y = 1.06x + 1.02; Placebo: R^2^ Linear = 0.100, y = 0.43x + 5.49. (**b**) Pre- and post-recording motor unit recruitment order in the infraspinatus muscle. Shape analysis threshold = 0.7 and amplitude margin = 30%. Capsaicin: R^2^ Linear = 0.335, y = 0.77x + 2.71; Placebo: R^2^ Linear = 0.090, y = 0.26x + 4.87.

The independent samples t-test for the difference in motor recruitment in the trapezius showed a significant effect (*p* = 0.033) when comparing the capsaicin group with the placebo group. The Kruskal–Wallis H test for the difference in motor recruitment in the infraspinatus did not show any significant effects (*p* = 0.123) between the capsaicin and placebo groups. Figures 6 and 7 provide a sample of results obtained that visualizes the recruitment order in the trapezius and infraspinatus respectively. If there was a perfect match in order between pre and post application then a line with slope of one would have been obtained.

### Intramuscular EMG

Table 2 shows the results of the Kruskal–Wallis H test. A significant difference in variance was seen between the difference of the post- and pre-recording variance when comparing capsaicin to the placebo condition. No significant difference was observed in the post- vs pre-recording variance in either the capsaicin or placebo condition.

## Discussion

The results using sEMG suggested that capsaicin induced sensitization is associated with a change in the motor unit recruitment pattern that violates Henneman`s size principle. Additionally, using iEMG, we demonstrated that there were changes in the characteristics of the first recruited motor unit with the application of capsaicin suggesting changes in anterior horn cell excitability that are likely related to the effects from the dorsal horn. Furthermore, we showed that these changes can occur many spinal levels from the spinal level associated with the site of application (T3-8), as evidenced by the statistical differences observed by both iEMG and sEMG recordings from the trapezius muscle (C3/4).

With regards to the pattern of recruitment of motor units, our results from the sEMG recordings of the trapezius muscle show that the capsaicin treatment was associated with motor units being recruited later as compared to the placebo ($$p=0.03)$$. This suggests that the capsaicin has some effect on a change in sequence of recruitment that is not consistent with the Henneman’s principle. This is observed in Fig. 6, as a large shift in the intercept for the capsaicin group (intercept = 7.88) as opposed to the placebo group (intercept = 1.74). Additionally, we observe a shift of the slope in the capsaicin group (slope = 0.72) as opposed to the placebo group (slope = 0.93). In the ideal case, the intercept and slope should be 0 and 1 respectively if Henneman’s principle is upheld. The observed results in the placebo group are fairly close to this ideal case whereas the capsaicin group show clear deviations from this ideal case.

These trends were not observed in the infraspinatus (Fig. 7a). There are a number of reasons as to why this may be the case. In many instances, the sEMG only identified a few motor units or, in some cases, no motor units were found within a subject. We believe that this was caused by very little activation of the infraspinatus muscle required for arm abduction or if the motor units were activated they fell below the sensitivity of the Delsys detection algorithm. Consequently, when calculating the average difference in recruitment, there were less matched pairs per subject compared to those seen in the trapezius. Therefore, when using a pre-set threshold of 0.8, there were not enough potentials available to conduct a robust statistical analysis. For this reason, we attempted a reanalysis using a slightly lower threshold of 0.7. When the threshold for the shape analysis was reduced (0.7 from 0.8) and the amplitude analysis margin increased (30% instead of 20%) allowing more potentials to be matched. This allowed the observation that the *trend* (Fig. 7b) is closer to that observed with the trapezius muscle (i.e. the slope of the capsaicin line is similar to that observed in the trapezius case). Based upon these analyses, we refrain from making any conclusions about the infraspinatus muscle and future research should involve an exercise that involves this muscle more directly. This can be achieved by performing an activity that specifically activates the infraspinatus muscle such as the side lying wiper exercise^39^.

Neuroglial changes due to sensitization that occur at the spinal cord segment(s) have been described^12,39,40^. Graven-Nielsen et al. noted that the spreading and sensitization from hyperalgesia is related to changes in descending control from supraspinal centers^41^. The influence of the nociception signalling from persistent periphery inputs has been described as well as the influences within the dorsal horn of the spinal cord^12,39^. It is becoming more evident that chronic pain conditions which are associated with sensitization affects the dorsal horn in the spinal cord^42^. It is also known from previous studies that capsaicin leads to activation of nociceptive afferent neurons and dorsal horn after central sensitization^23,42,43^. Additionally, activation of peripheral nociceptors by capsaicin can transitorily hyperactivate supraspinal areas including cortical supplementary motor area widely recognized to contribute to the control of movement^44^. The results reinforce Graven-Nielsen’s statement that hyperalgesia from central sensitization is related to changes in descending control from supraspinal centers^41^. It was also postulated that these abnormalities within the dorsal horn translate to changes in efferent pathways^45^. Fernandez-Carnero et al. demonstrated that intramuscular injection of glutamate into the infraspinatus latent myofascial trigger point caused an increase in electrical activity in the extensor carpi radialis brevis muscle^46^. The latter muscle is within the same neural segment as the infraspinatus muscle. Thus, an efferent change at the same spinal level was previously described, but an efferent effect at other spinal levels was not. Our study extends this research by examining how experimental sensitization can affect different spinal levels than the targeted one, as well as investigating these changes more microscopically, by focusing on individual motor units. We hypothesized that the neuroplasticity that was created by capsaicin application at the T3-8 levels caused a motor unit change as well as a change in the pattern of recruitment of the first few recruited motor units. This effect was from the afferent influence at T3-8 and recorded within the efferent portions of C3/4. Further research would be necessary to determine the nature of this influence. Potential causes could be excessive neurotransmitter production or neuroplasticity related neuronal influences at the other levels.

Our results using the intramuscular EMG recordings demonstrate that there was a statistically significant difference in the variability of the characteristics of individual motor units between the capsaicin and placebo treatment. This change in variability provides some insight into the mechanism of an ongoing barrage of nociceptive input produced by the application of capsaicin on efferent neurons and on motor unit. The motor unit is the composite of the activation of one anterior horn cell and the muscle fibers that it supplies^47^. In the present study, the calculation of variability changes took into account the entire motor unit potential, since template matching was used to define them. This would take into account aspects of the motor units potential’s attributes including amplitude, duration and area under the curve. The variability that we demonstrate suggests a change in excitability in the anterior horn cell responsible for that particular motor unit. Furthermore, we took into account the largest source of error, namely needle position change, between recordings. The statistical model we constructed allowed for this source of error to be accounted for as discussed in the “Limitations” section.

The other possibility is that the observed change in variability could also be due to the recruitment of a completely different motor unit. However, this is highly unlikely because (a) the pre- and post-recording motor units were matched based on their templates and (b) the statistical results have shown that there are no significant differences between post- and pre-recording variability for the capsaicin and placebo treatment only. Furthermore, at the time of acquisition the subject did not have any other stimuli and thus the likelihood that other excitatory effects were present is extremely low. For these reasons, we believe that the capsaicin resulted in the observed changes to the motor units.

### Limitations

While this work supports the possibility that motor unit recruitment patterns are changed and anterior horn cell excitability is changed, some factors may affect the results of this study.

In the sEMG recordings, the Delsys’ decomposition algorithm was assumed to be able to isolate individual motor units throughout a recording session providing the average templates of the MUAPs that they were able to track. To minimize the possible effects of this assumption, only tracked MUAPs with an algorithm reported 80% or greater confidence^37^ were used in our analyses. In addition, the number of detected matched pre-post motor units in the infraspinatus recordings was too low during the arm abduction activity. The infraspinatus is not the primary muscle involved in arm abduction, it is a shoulder stabilizer for this activity and is therefore minimaly activated. Consequently, as outlined above, the threshold was changed, but no conclusions were made about the results obtained from the infraspinatus muscle. An increase in sample size will be needed along with using an activity that adequately activates both muscles to confirm that the infraspinatus muscle results in similar conclusions to the trapezius.

For the iEMG recordings, needle movement is the largest source of possible error and could have affected the variability in the signals observed. This was accounted for by subtracting the overall effects of capsaicin from placebo. In other words: (post-capsaicin + post-needle position + noise) from (pre-capsaicin + pre-needle position + noise) and also (post-placebo + post-needle position + noise) from (pre-placebo + pre-needle position + noise). Therefore, the effect observed would be related to the experimental intervention (capsaicin) only, as the needle position in both capsaicin and placebo group should be random. Since both within capsaicin and within placebo show no significant results, but the between the difference of the post- and pre-recording signal between capsaicin and placebo was significant, this shows that the variability difference can be attributed to the application of capsaicin alone.

In addition, most participants were under the age of 55 and while we do not expect the trends seen here to be different, further investigation is needed to observe if these results are reflective in an older population.

## Conclusions

We demonstrate that experimental central sensitization induced by topical capsaicin creates abnormalities on the motor unit level increasing the excitability of individual motor units and changing the motor unit recruitment patterns, thereby violating the Henneman’s size principle. Furthermore, the observed changes on motor unit level measured by sEMG and iEMG was not restricted to the same location supplied by afferent nerve fibers of capsaicin application. This suggests that central abnormalities caused by topical capsaicin can traverse spinal levels modulating motor outputs. This may provide insights for the neurophysiological influence of central sensitization on changes in efferent responses and movement patterns that occur in patients with persistent pain. However, the clinical implication of this experimental findings on the movement properties needs to be further investigated.

## Supplementary Information

Supplementary Information.
