# Supplementary material for: A randomized double blinded placebo controlled study to evaluate motor unit abnormalities after experimentally induced sensitization using capsaicin
Source: Sci Rep. 2021 Jul 2;11:13793. doi: 10.1038/s41598-021-93188-7 (PMC8253857; doi:10.1038/s41598-021-93188-7)
Supplement: Supplementary file 1 — Supplementary Information. [file 41598_2021_93188_MOESM1_ESM.docx]

# Evaluation of Motor Unit Abnormalities after Experimentally Induced Sensitization Using Capsaicin: A Randomized, Double-Blinded, Placebo-Controlled Study

# Valerie Evans^1,2,†^, Ryan G. L. Koh^1,†^, Felipe C. K. Duarte^3^, Lukas Linde^4,5^, Mohammadreza Amiri^1^, Dinesh Kumbhare^1,2,6*^

^1^Toronto Rehabilitation Institute, University Health Network, Toronto, ON, Canada

^2^Institute of Biomaterials and Biomedical Engineering, University of Toronto, Toronto, ON, Canada

^3^Division of Research and Innovation, Canadian Memorial of Chiropractic College, Toronto, ON, Canada

^4^International Collaboration On Repair Discoveries, University of British Columbia, Vancouver, BC, Canada

^5^School of Kinesiology, University of British Columbia, Vancouver, BC, Canada

^6^Division of Physical Medicine & Rehabilitation, Department of Medicine, University of Toronto, Toronto, ON, Canada

^†^The first and second authors contributed equally

**APPENDIX A**

| **Algorithm 1:** Automated Motor Unit Matching method | |
| --- | --- |
| **Input:** Matrix of averaged values from shape and amplitude analysis | |
| **Output: M**atched pre-post motor units | |
| 1: | **loop** through each pre-motor unit (row) and each post-motor unit (col) |
| 2: | **if** value at current location is the highest in that row and column, **and** the number is not equal to zero |
| 3: | **Record** match between the pre- and post- motor unit, and set the row and col |
|  | to 0 (i.e. remove the pre- and post- motor unit from further pairing) |
| 4: | Return matched pre-post motor unit pairs |
|  |  |
|  |  |

**APPENDIX B**

Average Difference per person (for T test USE):

$$={Average\left( Differences \right)}_{per person}$$

$$=\frac{\left( 8 \right)+\left( 6 \right)+\left( 9 \right)+\left( 11 \right)+\left( 9 \right)+\left( 11 \right)+\left( 4 \right)}{7}$$

= 8.286

| MUPRE | MUPOST | Difference Array |
| --- | --- | --- |
| 1 | 9 | 8 |
| 2 | 8 | 6 |
| 4 | 13 | 9 |
| 5 | 16 | 11 |
| 6 | 15 | 9 |
| 8 | 19 | 11 |
| 14 | 18 | 4 |

Supplementary Figure 1: Participant 11 Pre/Post Difference

In order to compare the reorganization in recruitment, the difference in order of recruitment was calculated and put into an array. This difference array was calculated for each person, and an average difference in recruitment per person was analysed using a t-test. The reason for having an average per person was to avoid any bias by having multiple data points per person that correspond to different motor units. Assumptions of normality and homogeneity of variances were verified from the Shapiro- Wilk test (p=0.187) and Levenes Statistic (p=0.081), therefore a t-test can be used.
